# Validation and Recalibration of Two Multivariable Prognostic Models for Survival and Independence in Acute Stroke

**DOI:** 10.1371/journal.pone.0153527

**Published:** 2016-05-26

**Authors:** Julius Sim, Lucy Teece, Martin S. Dennis, Christine Roffe, SOࠢS Study Team

**Affiliations:** 1 Institute for Primary Care and Health Sciences, Keele University, Keele, United Kingdom; 2 Centre for Clinical Brain Sciences, University of Edinburgh, Edinburgh, United Kingdom; 3 Stroke Research in Stoke, University Hospitals of North Midlands NHS Trust, Stoke-on-Trent, United Kingdom; 4 Institute for Science and Technology in Medicine, Keele University, Keele, United Kingdom; University of Perugia, ITALY

## Abstract

**Introduction:**

Various prognostic models have been developed for acute stroke, including one based on age and five binary variables (‘six simple variables’ model; SSVMod) and one based on age plus scores on the National Institutes of Health Stroke Scale (NIHSSMod). The aims of this study were to externally validate and recalibrate these models, and to compare their predictive ability in relation to both survival and independence.

**Methods:**

Data from a large clinical trial of oxygen therapy (*n* = 8003) were used to determine the discrimination and calibration of the models, using C-statistics, calibration plots, and Hosmer-Lemeshow statistics. Methods of recalibration in the large and logistic recalibration were used to update the models.

**Results:**

For discrimination, both models functioned better for survival (C-statistics between .802 and .837) than for independence (C-statistics between .725 and .735). Both models showed slight shortcomings with regard to calibration, over-predicting survival and under-predicting independence; the NIHSSMod performed slightly better than the SSVMod. For the most part, there were only minor differences between ischaemic and haemorrhagic strokes. Logistic recalibration successfully updated the models for a clinical trial population.

**Conclusions:**

Both prognostic models performed well overall in a clinical trial population. The choice between them is probably better based on clinical and practical considerations than on statistical considerations.

## Introduction

There is current interest in the development and testing of prognostic models in acute stroke. Such models provide estimates of outcome for individual patients based on a number of predictors [1]. They may also be useful in audit and resource allocation at the service level. In research, prognostic models may provide important epidemiological data, and may be used to determine case-mix and/or subgrouping in intervention studies [2].

Prognostic models in stroke are characteristically based upon information on the individual’s health and functional capacity at the time of–or immediately preceding–the stroke and clinimetric measures indicating stroke severity [3,4]. A well-performing prognostic model should exhibit discrimination, calibration, parsimony and practicality (see Box 1 for definitions).

**Box 1. Desirable properties of prognostic models**

*Discrimination* (accuracy of classification): The model should correctly classify individuals in terms of a positive (e.g. functional recovery) or negative (e.g. death) future state, and thereby exhibit high levels of sensitivity and specificity [5].

*Calibration* (accurate estimates of risk): The model should correctly predict, for a given risk, the proportion of patients who will achieve a specified future state. This is achieved by determining how well the probabilities derived from the model agree with observed outcomes [5].

*Parsimony*: The model should achieve good discrimination and calibration on the basis of a manageable quantity of information derived from data likely to be available for most, or preferably all, patients [6].

*Practicality*: The model should permit easy calculation of prognostic risk, especially if used in routine clinical practice, and be easy to interpret [7].

Teale et al [8] reviewed 17 externally validated prognostic models in acute stroke. Methodological weaknesses were identified in a number of these models, and some lacked appropriate validation in independent samples. Two models that were found to perform well are the six simple variables model (SSVMod) [9] and the NIHSS + age model (NIHSSMod) [10].

### The prognostic models

The SSVMod was developed in data from the Oxford Community Stroke Project, a community-based incidence study of first-ever stroke [9], and is based upon six variables: age, as a continuous variable, and five binary variables, coded yes/no (living alone; independent pre-stroke; normal Glasgow Coma Scale verbal score [4]; able to lift both arms against gravity; able to walk unaided). Patients on whom the SSVMod was developed were assessed at a median of 4 days post stroke. Predictions can be derived for 30-day survival and for independence at 6 months.

The more recent NIHSSMod was developed from a database in the Stiftung Deutsche Schlaganfall-Hilfe, a data bank representing a hospital-based cohort [10], and is based on two variables: age and the National Institutes of Health Stroke Scale (NIHSS) [3], which provides a single score for stroke severity based on 13 items. Patients were assessed on the NIHSS within 6 hours of the stroke. This model allows probabilities to be calculated for both death and dependence (operationalized in the model as ‘incomplete recovery’) at 100 days post stroke. Table 1 gives details of the models.

**Table 1:** Details of the original predictive models [9,11].

Model	Coefficients							
	Constant	NIHSS	Age	Living alone: yes	Independent pre-stroke: yes	Normal GCS verbal score; yes	Able to life both arms against gravity; yes	Able to walk unaided; yes
SSVMod–survival*	– ^‡^	–	multiply by 0.034	– 0.406	+ 0.501	+ 0.766	+ 0.851	+ 0.489
SSVMod—independence^†^	12.340	–	multiply by –0.051	+ 0.661	– 2.744	– 2.160	– 2.106	– 1.311
NIHSSMod—mortality^†^	– 7.040	multiply by 0.155	multiply by 0.049	–	–	–	–	–
NIHSSMod—independence^†^	– 5.782	multiply by 0.272	multiply by 0.049	–	–	–	–	–

* Cox survival model

^†^ Logistic regression model

^‡^ Baseline survival at 30 days = 0.631

Both these models have been individually validated in several studies [11–16]; see Table 2. However, their relative performance has received little attention. A recent study [16] compared the NIHSSMod with the SSVMod and an adaptation of the SSVMod in which one variable (the score for living alone) was omitted, in relation to independence at 3 and 12 months. The NIHSSMod produced measures of calibration slightly superior to those of the SSVMod (though not significantly). The models performed comparably in patients with haemorrhagic versus ischaemic strokes [16]. No attempt to recalibrate the models (i.e. to adjust their coefficients) appears to have been made hitherto.

**Table 2:** Characteristics of previous validation studies.

Study	Population	Type of stroke presentation	Clinical presentation	Model(s) validated
Counsell et al [11]	Trial patients (n = 2955). Mean age 73 years, 50% male	Any.	89% independent before stroke.	SSVMod
Reid et al [12]	Hospital-based stroke register (*n* = 538). Median age 74 yrs, 53% male.	Hyperacute stroke (87% ischaemic).	Median stroke severity 6 out of 10.* 81% independent before stroke.	SSVMod
König et al [13]	Trial patients (*n* = 5419). Mean age 69, 59% male.	Ischaemic.	Mean NIHSS score 13.4. 44% with Barthel Index (0–100 version) score ≥ 95.	NIHSSMod
SCOPE [14]	Trial patients (*n* = 537). Mean age 74 yrs, 54% male.	Hyperacute ischaemic stroke (21% had had previous stroke).	97% independent before stroke.	SSVMod
Teale et al [15]	Hospital-based cohort study (*n* = 176). Mean age 73, 53% male.	Transient ischaemic attack excluded; otherwise unspecified.	Median Barthel Index (0–20 version) score 17	SSVMod
Ayis et al [16]	Hospital-based (mainly) stroke registers across countries (*n* = 2033). Mean age 71 years, 52% male.	First strokes only (82% ischaemic).	Median stroke severity ‘moderate’. ^†^ 90% independent before stroke. ^‡^	SSVMod and NIHSSMod

* High scores indicate greater severity

^†^ Based on NIHSS score between 5 and 14

^‡^ Based on Barthel Index (0–20 version) score between 12 and 20.

### Aims

The current study therefore sought to evaluate further the discrimination and calibration of the SSVMod and the NIHSSMod in a cohort of stroke patients from a randomized controlled trial. Specific aims were to:

- determine the external validity of the models by comparing their performance in an external data set different from those of the original derivation studies.
- compare their performance in subgroups of patients with either ischaemic or haemorrhagic strokes.
- compare the predictive ability of the models and their generalizability to timepoints other than those on which they were developed.
- recalibrate the models in a clinical trial population.

## Methods

### Data

The models were validated in a cohort of patients from the Stroke Oxygen Study [17], a large (*n* = 8003) randomized trial of oxygen therapy in hospitalized patients with acute stroke, recruited in 136 collaborating centres in the UK between 2008 and 2013. The inclusion criteria for the trial were that patients must have had a stroke within the preceding 24 hours and have no definite indications for, or definite contraindications against, oxygen therapy. Exclusion criteria were patients with other serious life-threatening conditions likely to lead to death within the following few months (who would, therefore, be unlikely to benefit from oxygen treatment), or patients in whom stroke was not the main clinical problem. Patients were treated in the first 72 hours with either continuous oxygen, nocturnal oxygen, or no oxygen. We analysed the 8003 patients who had reached at least the three-month outcome point–with no missing values in respect of the predictor variables in the models–at the time of the present study. Subgroups of patients with either ischaemic (*n* = 6369) or haemorrhagic (*n* = 559) strokes were identified (these do not comprise the total study sample, as the nature of 1075 patients’ stroke was undetermined).

The outcomes of independence and incomplete recovery were defined in relation to a score <3 on the Oxford Handicap Scale (OHS) [18] and a score >95 on the Barthel Index (0–100 version)[19], respectively–as per the original studies [9,10]. To make the models comparable, probabilities for death and incomplete recovery were converted to those for survival and complete recovery, respectively. The modified Rankin Scale (mRS) was used in the validation sample as a proxy for the OHS, as the relevant cutoffs are equivalent [18]. Follow-up data on the mRS were collected by post, or by telephone in the case of non-responders.

Table 3 defines the outcomes against which the models were tested. The timepoints at which outcomes were assessed differed in one respect from those for which the models were developed–the NIHSSMod was assessed at 100 days rather than 6 months. The data used for the testing of the models is in S1 Data.

**Table 3:** Characteristics of the prognostic models.

	Outcome 1: survival		Outcome 2: independence	
	SSVMod	NIHSSMod	SSVMod	NIHSSMod
Probability given by model	Survival	Death*	Independence	Incomplete recovery^†^
Scale for outcome	Binary	Binary	Oxford Handicap Scale (< 3)^‡^	Barthel index (< 95)
Time of prediction	30 days	100 days	6 months	100 days
Statistical method used	Cox regression	Logistic regression	Logistic regression	Logistic regression

* converted to survival for analysis

^†^ converted to complete recovery for analysis

^‡^ equivalent point on the modified Rankin Scale was used in analysis

### Validation methods

The prognostic characteristics of the models to be tested were *discrimination* (the ability of the model to distinguish participants with the outcome from those without) and *calibration* (the extent to which outcomes predicted by the model in specified risk-defined subgroups are similar to those observed in the validation dataset) [5]. Statistical analysis was performed in Stata 13.

The discrimination of the models was assessed using the concordance (C) statistic; for binary outcomes this is equivalent to the area under the receiver operating characteristic (ROC) curve [20], which plots sensitivity against 1 minus specificity. The C-statistic normally ranges from .5 to 1, with a value of 1 representing perfect discrimination and a value of .5 representing discrimination no better than chance. C-statistics were determined in relation to the observed binary outcomes (survived/died; independent/dependent) at the relevant timepoints. The difference between the C-statistics estimated for the models was tested statistically for each outcome [21].

The calibration of the models was displayed using calibration plots, which plot the model predictions against grouped observations in the data. For a well-calibrated model, the plotted markers should lie on or near the diagonal reference line. Calibration in the large (mean calibration) of the models was tested by comparing the observed and predicted outcomes of the model in a logistic regression model, with the risk score as an offset variable; a non-significant difference between predicted and observed outcomes indicates good calibration. Calibration was further tested by using a Hosmer-Lemeshow (HL) goodness of fit statistic, which compares observed and predicted outcomes in groups of patients. More than one method of grouping is recommended [20], and the HL statistic was therefore calculated in relation to deciles of risk, deciles of patient numbers, and the maximum number of groups (up to 100, with group size ≥ 5). A non-significant HL test indicates good calibration. The magnitude of miscalibration was calculated as the calibration slope. This is the regression slope of the linear predictor, and the closer the slope coefficient is to 1, the better the calibration [22]. A slope below 1 may indicate unduly extreme predictions (i.e. low predictions were too low and high predictions were too high) whereas a slope greater than 1 may indicate that predictions do not vary sufficiently (i.e. predicted risks are too low)[23,24].

### Model recalibration

The models were updated by a process of recalibration. First, the models were updated using recalibration in the large, which adjusts the average predicted probability so that it equals the observed event rate. This method can be applied when a difference in the outcome incidence is suspected [25]. Second, the models were updated by logistic recalibration [26], which corrects the mean calibration and adjusts the regression coefficients of the predictor by a single adjustment factor. This method can be applied when the coefficients of the original model may have been over-fitted; it assumes the relative effects of the predictors are similar but allows the predictors to have a larger or smaller effect. See Appendix for details of these methods.

### Ethics

The Stroke Oxygen Study received approval from the North Staffordshire Research Ethics Committee on 24^th^ January 2007 (COREC 06/Q2604/109). Written informed consent was obtained from all participants where possible. For patients not competent to give written consent at the time of enrolment, assent was obtained from a relative or an independent physician and full informed consent was obtained from the patient when he or she was competent to give it. These patients were not excluded from the trial as a considerable proportion of acute stroke patients will have receptive and/or expressive problems and it was considered important for the trial results to be generalizable to these patients. The Ethics Committee approved this consent procedure.

## Results

Demographic characteristics of the original and the validation samples are shown in Table 4. The age of patients in the validation sample was similar to that of patients in the SSVMod development sample, but somewhat higher than that of patients in the NIHSSMod development sample. Sex distribution also differs, with a higher proportion of males in the validation sample than in the SSVMod development sample but a smaller proportion than in the NIHSSMod development sample. The median (interquartile range [IQR]) SSVMod risk scores for independence and survival in the validation sample were .427 (.120, .712) and .921 (.859, .955), respectively. The median (interquartile range) NIHSSMod risk scores in the validation sample for incomplete recovery and death were .316 (.174, .603) and .071 (.040, .135), respectively. Ninety-two percent of patients in the validation sample were independent prior to their stroke. Twenty-six percent were able to walk unaided at the time of randomization.

**Table 4:** Demographic characteristics of the original and validation samples.

	Outcome 1: survival		Outcome 2: independence	
	SSVMod	NIHSSMod	SSVMod	NIHSSMod
Age in original sample, years; mean (SD)	73 (12)	67 (12)	73 (12)	67 (12)
Age in validation sample, years; mean (SD)	72 (13)	72 (13)	71 (13)	71 (13)
Sex in original sample; % male	48	61	48	61
Sex in validation sample; % male	55	55	56	57

Denominators for survival: 8003 for both models. Denominators for independence: 5667 for SSVMod, 5373 for NIHSSMod)

### Discrimination

**Fig 1:**
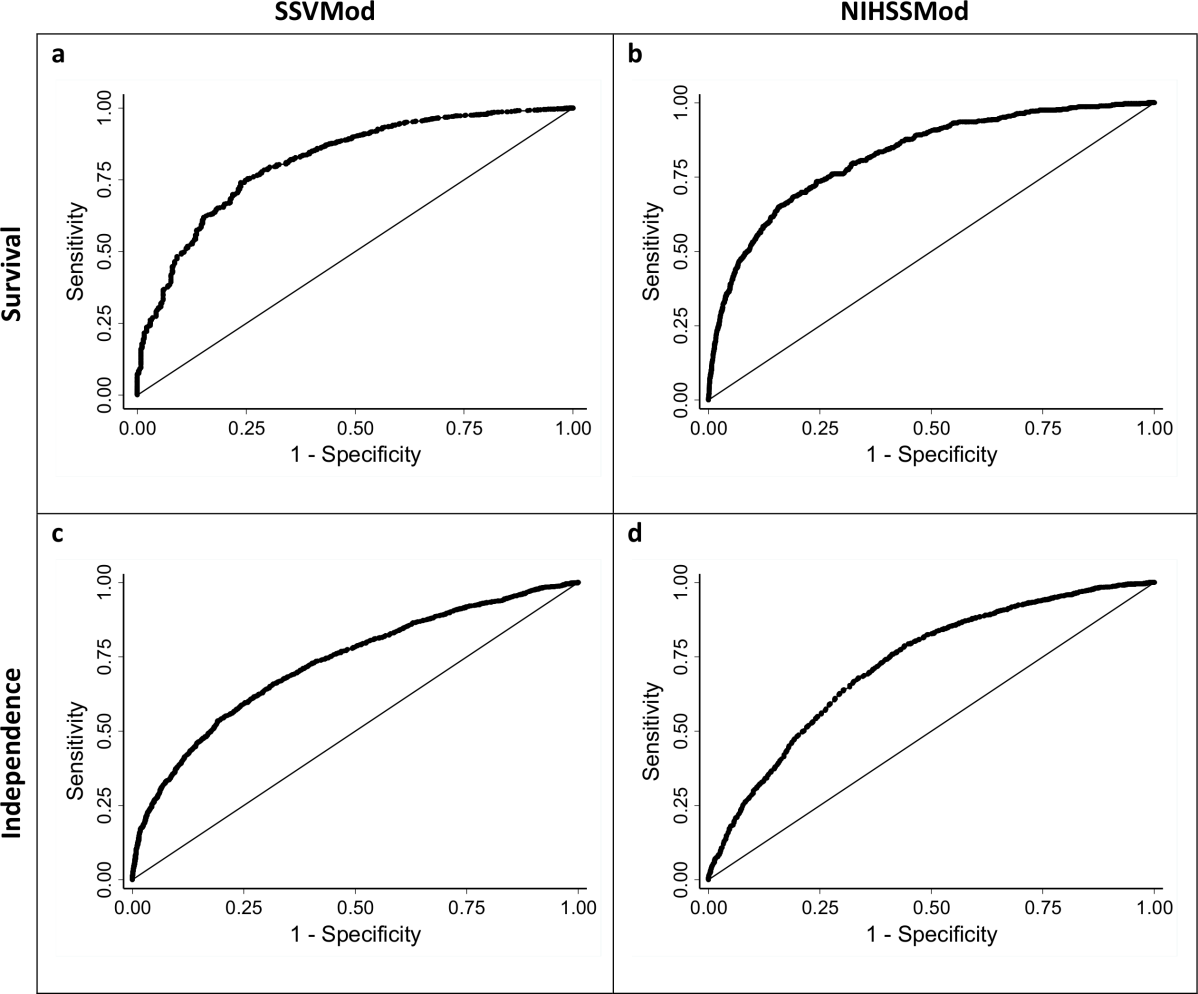
Receiver operating characteristics (ROC) curves for survival and independence, for the total sample. Optimally, the curve should lie towards the upper left corner of the plot. Survival: assessed at 30 days for the SSVMod and at 100 days for the NIHSSMod. Independence: assessed at 6 months for the SSVMod and at 3 months for the NIHSSMod.

Fig 1 shows the ROC curves for survival and independence for the SSVMod (plots *a* and *c*) and the NIHSSMod (plots *b* and *d*), for the whole sample. Table 5 shows the corresponding C-statistics and tests for difference for the total sample, and Table 6 shows the corresponding information for the subgroups. Overall, C-statistics for survival exceed those for independence, indicating better discrimination. In the subgroups, other than for 30-day survival, discrimination appears slightly worse for haemorrhagic than for ischaemic strokes. For 6-month independence, C-statistics from the NIHSSMod are larger than those from the SSVMod, except among haemorrhagic strokes, whereas for 3-month independence C-statistics from the SSVMod exceed those from the NIHSSMod; the C-statistic for the NIHSSMod among haemorrhagic strokes is particularly low at .684. Each model therefore discriminates somewhat better with respect to its ‘own’ outcome, though it should be noted that a number of the differences in C-statistics were non-significant. Discrimination also tends to be better for earlier than for later outcomes.

**Table 5:** C-statistics for survival and independence.

	SSVMod			NIHSSMod			Test for equality
	C-statistic	95% CI	*n*	C-statistic	95% CI	*n*	*p* value
**30-day survival**							
total	.816	.794, .838	8003	.837	.815, .858	8003	.001
**100-day survival**							
total	.802	.784, .820	8003	.823	.806, .840	8003	< .001
**3-month-independence**							
total (1)	—	—	—	.728	.714, .741	5373	—
total (2)	.735	.722, .748	5373	.728	.714, .741	5373	.160
**6-month independence**							
total (1)	.725	.712, .739	5667	—	—	—	—
total (2)	.725	.712, .739	5667	.731	.718, .745	5667	.242

(1) Estimate using all available data

(2) Estimate using pairwise deletion. CI = confidence interval.

**Table 6:** C-statistics for survival and independence in subgroups defined by type of stroke (ischaemic and haemorrhagic). The *p* values from the tests for equality of the C-statistics should be interpreted with regard to the differing denominators of ischaemic and haemorrhagic strokes.

	SSVMod			NIHSSMod			Test for equality
	C-statistic	95% CI	*n*	C-statistic	95% CI	*n*	*p* value
**30-day survival**							
ischaemic	.815	.791, .840	6369	.834	.809, .858	6369	.008
haemorrhagic	.809	.714, .904	559	.838	.749, .928	559	.109
**100-day survival**							
ischaemic	.805	.785, .825	6369	.823	.804, .843	6369	.002
haemorrhagic	.788	.721, .855	559	.818	.751, .885	559	.066
**6-month independence**							
ischaemic	.730	.715, .745	4546	.737	.722, .752	4546	.252
haemorrhagic	.722	.670, .774	398	.710	.657, .763	398	.523
**3-month independence**							
ischaemic	.741	.726, .756	4318	.735	.720, .750	4318	.310
haemorrhagic	.712	.660, .765	373	.684	.630, .738	373	.148

CI = confidence interval

### Calibration

Calibration in the large for the two outcomes is shown for each model in Table 7. Both models under-predicted the number of patients surviving at 30 and 100 days and over-predicted the number independent at 3 (NIHSSMod) and 6 months (SSVMod); the *p* values from the logistic regression test indicate that the discrepancy was not, however, significant in respect of independence in relation to the SSVMod. The figures in Table 7 for ischaemic and haemorrhagic stroke are similar.

**Table 7:** Calibration in the large of the models. Data are counts (%); *p* values are derived from a logistic regression model. Figures are given for all patients and separately for those with ischaemic and haemorrhagic strokes.

	SSVMod			NIHSSMod		
	Observed	Expected	*p* value	Observed	Expected	*p* value
**Survival**						
total	7628 (95.3)	7068 (88.3)	<0.001	7378 (92.2)	7000 (87.5)	< .001
ischaemic	6066 (95.2)	5263 (82.6)	<0.001	5867 (92.1)	5568 (87.4)	< .001
haemorrhagic	531 (95.0)	494 (88.4)	<0.001	511 (91.4)	489 (87.5)	< .001
**Independence**						
total	3422 (60.4)	2659 (46.9)	0.160	2685 (50.0)	2001 (37.2)	< .001
ischaemic	2733 (60.1)	2137 (47.0)	0.098	2146 (49.7)	1607 (37.2)	< .001
haemorrhagic	239 (60.1)	189 (47.5)	0.562	185 (49.6)	132 (35.4)	< .001

Survival: assessed at 30 days for the SSVMod and at 100 days for the NIHSSMod. Independence: assessed at 6 months for the SSVMod and at 3 months for the NIHSSMod. Denominators for survival: 8003 for both models (6369 for ischaemic, 559 for haemorrhagic). Denominators for independence: 5667 for SSVMod (4546 for ischaemic, 398 for haemorrhagic); 5373 for NIHSSMod (4318 for ischaemic, 373 for haemorrhagic).

In relation to both survival and independence, the HL test was significant, for each method of grouping, in relation to both the SSVMod and the NIHSSMod model (data not shown). The calibration slope for survival was 1.308 for the SSVMod and 0.975 for the NIHSSMod. For independence, the calibration slope was 0.470 for the SSVMod and 0.629 for the NIHSSMod. The slopes are closer to 1 for survival than for independence, suggesting superior calibration.

**Fig 2:**
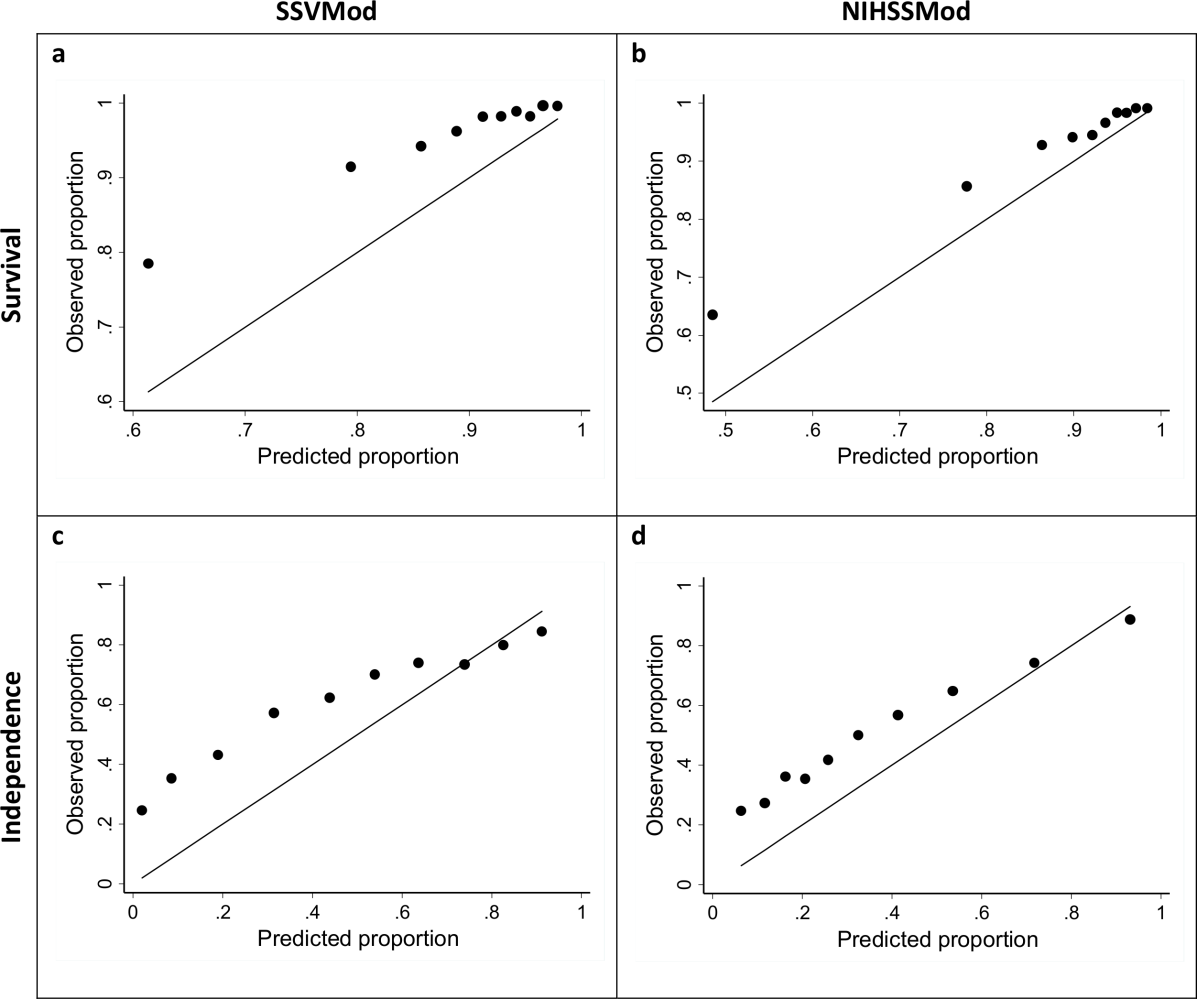
Calibration plots for survival and independence, for the total sample and based on deciles of patient numbers. Survival: assessed at 30 days for the SSVMod and at 100 days for the NIHSSMod. Independence: assessed at 6 months for the SSVMod and at 3 months for the NIHSSMod. For illustrative clarity, the origins for the axes vary between plots.

Calibration plots are shown in Fig 2; as the methods of grouping produced similar results in the HL test, plots are shown just for deciles of patient numbers. Judged by the approximation of the plotted markers to the diagonal, the NIHSSMod (plots *b* and *d*) appears to show better calibration than the SSVMod (plots *a* and *c*), for both outcomes.

### Model recalibration

Table 8 shows the results for the recalibration of the two models, using both recalibration in the large (updating the intercept) and logistic recalibration (updating the intercept and the slope; see Appendix). Logistic recalibration produced models with good calibration, confirmed by a non-significant HL test. However, recalibration in the large only gave a non-significant HL test for the NIHSSMod for 100-day survival.

**Table 8:** Recalibration of the SSVMod and the NIHSSMod.

Model and outcome	Recalibrated parameter estimates		Hosmer-Lemeshow test (*p* values)		
	Recalibration in the large	Logistic recalibration	Original	Recalibration in the large	Logistic recalibration
SSVMod: 30-day survival	0.813^e(LP)^	0.813^e(1.308 x LP)^	< .001	< .001	.509
NIHSSMod: 100-day survival	–0.655 + LP	–0.688 + (0.975 x LP)	< .001	.346	.307
SSVMod: 6-month independence	0.869 + LP	0.628 + (0.470 x LP)	< .001	< .001	.239
NIHSSMod: 100-day independence	0.735 + LP	0.425 + (0.629 x LP)	< .001	< .001	.155

LP = original linear predictor equation (see also Table 1)

## Discussion

This study sought to perform a comparative validation of the SSVMod and the NIHSSMod by examining their discrimination and calibration in an external dataset derived from a large randomized trial; these characteristics of the models were also tested in subgroups of patients with either ischaemic or haemorrhagic strokes. Additionally, the two models were updated in the context of a clinical trial population.

In this study, discrimination was somewhat better for survival than for independence for both models; however, higher discrimination may be anticipated for outcomes such as death that are measured without error and for which predictors are often easier to identify. Additionally, discrimination tended to be better for earlier than for later outcomes, probably because there is less likelihood of intervening events that may influence outcome. There are some differences between ischaemic and haemorrhagic strokes. Haemorrhagic strokes are often fatal, largely irrespective of the patient’s age. Patients with ischaemic strokes, even severe ones, are more likely to die of complications than as a result of the stroke, and such complications may be more likely in older patients. Both of these considerations suggest that prognostic models incorporating age may perform better in ischaemic than in haemorrhage strokes. The NIHSSMod shows noticeably lower discrimination than the SSVMod for haemorrhagic strokes in relation to 3-month independence. However, *p* values for the mean calibrations and the comparisons between C-statistics must be interpreted with caution, owing to the different denominators in these comparisons.

Both the prognostic models showed shortcomings with regard to calibration, tending to over-predict survival and under-predict independence. This may partly reflect the eligibility criteria of the RCT sample–in which, for example, moribund patients were not included–and improvements in care (e.g. due to thrombolysis and care in specialized stroke units) since the models were first developed. Also relevant is that the development cohort for the NIHSSMod [10] excluded patients with pre-existing disability (mRS score ≥3), and the cohort for the SSVMod [9] is likely to have excluded early deaths by virtue of collecting data for the model at a median of 5 days after stroke, in contrast to within 24 hours of stroke onset in the validation cohort. Accordingly, both models might be expected to give different predictions of survival (and of independence in the case of the NIHSSMod) in our validation cohort. However, well-calibrated models will fail a statistical test if the sample is large, owing to increased statistical power. As was observed for discrimination, calibration, as judged by the calibration slopes, was better for survival than for independence, and similar factors to those suggested in the case of discrimination are likely to explain this. Reflecting the relative magnitudes of the calibration slopes, the calibration plots indicate that the NIHSSMod performs slightly better than the SSVMod. There is, however, little difference in the calibration of the models between ischaemic and haemorrhagic strokes, reflecting earlier findings [16].

In comparison with other studies, the C-statistics calculated as a measure of discrimination for 100-day survival were lower than those reported by Ayis et al [16] for 3-month survival (.80 vs .90 for the SSVMod, .82 vs .88 for the NIHSSMod). This might appear to reflect the longer prediction time in our study, except that the C-statistics for 30-day survival were also lower than Ayis et al’s figures for 3-month survival. A more plausible explanation is that participants from a clinical trial are likely to be more homogeneous than patients in a community or general clinical population, making discrimination more difficult. Furthermore, independence was defined by Ayis et al as a score ≥12 on the Barthel Index (0–20 version), rather than in terms of the mRS, as in our validation study. In relation to the NIHSSMod, the C-statistic for 100-day survival was higher in our study than in König et al’s study [13] (.82 vs .71) but that for 3-month independence as lower (.73 vs .81). This may reflect differences in the two cohorts–König et al’s patients were somewhat younger than those in our cohort (69 vs 72), but with a higher mean NIHSS score (13 vs 7). For the SSVMod, the C-statistic for 30-day survival was higher than that reported by SCOPE [14] (.82 vs .73). In contrast, the C-statistic for 6-month independence (.73) was lower than those reported by SCOPE (.82) [14] and Reid et al (.79) [12]. The calibration plot for 30-day survival appears to be worse than that reported by SCOPE [14], but the plots for 90-day independence are similar. Differences vis-à-vis the SCOPE study may again reflect differences in the patient population–the SCOPE study included almost exclusively patients who were independent before stroke. In addition, we used the mRS as a proxy for the OHS, which was utilized in the SCOPE study. Whilst the meaning of the relevant cutoff (<3) is equivalent in the two scales, the cutoff is described using somewhat different wording [18], which may account for some of the difference in findings.

Although there is some indication that the NIHSSMod performs better than the SSVMod in terms of calibration and, for survival, in terms of discrimination, differences between the models are generally small and the small *p* values reflect the large sample size; it is therefore hard to reach a conclusive judgment regarding the relative predictive power of the two models. It is likely that judgments as to the relative utility of the two models should instead be related to clinical and practical considerations. The SSVMod requires information on a small number of variables, whereas the NIHSS is a multi-item scale requiring a degree of training [27]–though the NIHSS is becoming a standard method of clinical assessment in acute stroke and nomograms and computer programs exist for both the SSVMod and the NIHSSMod. The NIHSS scores a person’s current performance and has to be undertaken as part of a clinical examination. In contrast, the SSVMod is a combination of aspects of physical performance and the history readily collected from clinical records or by interview. Information for the SSVMod may therefore be quicker and easier to collect. Notwithstanding this, in the UK, the NIHSS is collected as a matter of standard practice as part of the Sentinel Stroke National Audit Programme (SSNAP; https://www.rcplondon.ac.uk/projects/sentinel-stroke-national-audit-programme). The NIHSSMod, unlike the SSVMod, does not reflect pre-stroke status; this may not be problematic in trial populations, as patients who are dependent pre-stroke are excluded in most trials, but it may be less appropriate for everyday clinical populations, where pre-stroke dependence may be a more important predictive factor.

It has been recommended that, rather than developing new models *ab initio*, researchers should look at recalibrating existing models [1]. In our study, updating of the models in the context of a clinical trial population using recalibration in the large was successful for the NIHSSMod in respect of 100-day survival, but not for the other model/outcome combinations, where the significant HL tests indicate inadequate calibration. In contrast, logistic recalibration was successful for all model/outcome combinations. As recalibration in the large only updates the average predicted risk, this was expected to be less effective than logistic recalibration. The logistic recalibration thereby provided updated models suitable for a clinical trial population. Extrapolation to clinical populations is less certain, given that they are commonly more heterogeneous than trial populations–nonetheless, the broad inclusion criteria and minimal exclusion criteria for the trial, and the fact that the intervention tested within the trial is commonly used in clinical practice in the UK [28], suggest that the recalibrated models are likely to have some broader clinical relevance.

## Appendix

### Details of the methods of model updating

Recalibration in the large. This method is used in cases where a difference in the outcome incidence is suspected [26]. For the logistic regression models this was achieved by fitting a model with only one free parameter, and with an offset variable equal to the linear predictor of the original model. The models were updated by adding the coefficient of the free parameter to the linear predictor; the individual risks were then recalculated:

recalibrated linear predictor=updated intercept+original linear predictor

For the survival analysis model recalibration in the large was accomplished by fitting a Cox proportional hazards model with the linear predictor as the only parameter and estimating the baseline survival at 30 days, setting the linear predictor equal to 0. The individual risks were recalculated by replacing the current baseline survival in the prognostic model with the updated value:

$$\text{recalibrated linear predictor}={\text{updated baseline survival}}^{e\left(\text{original linear predictor}\right)}$$

Logistic recalibration. This method is used in cases where the coefficients of the original model may have been over-fitted; it assumes similar *relative* effects of the predictors but allows for a larger or smaller *absolute* effect of the predictors [26]. For the logistic regression models this was achieved by fitting a model with the linear predictor of the original model as a single predictor. The models were then updated by multiplying the linear predictor by the coefficient and adding the estimated intercept; the individual risks were then recalculated:

recalibrated linear predictor=updated intercept+(coefficient x original linear predictor)

For the survival analysis model this was accomplished by fitting a Cox proportional hazards model with the linear predictor as the only parameter and estimating the baseline survival at 30 days with the linear predictor equal to 0. The model was then updated by replacing the current baseline survival value for the updated one and multiplying the linear predictor by the coefficient in the model; the individual risks were then recalculated:

$$\text{recalibrated linear predictor}={\text{updated baseline survival}}^{e\left(\text{coefficient x original linear predictor}\right)}$$

## Supporting Information

S1 Data Data for the testing of the prognostic models. (DTA)
